# Correction to “Pooled analyses of randomized controlled trials on pyrotinib plus capecitabine and a rethink of the first‐line options for HER2‐positive relapsed or metastatic breast cancer”

**DOI:** 10.1002/cai2.69

**Published:** 2023-04-11

Guan X, Ma F, Xu B. Pooled analyses of randomized controlled trials on pyrotinib plus capecitabine and a rethink of the first‐line options for HER2‐positive relapsed or metastatic breast cancer. Cancer Innovation.2022;1‐5.

In the funding information, the Grant/Award Number of National Nature Science Foundation of China was incorrect. The Grant/Award Number should be “82103634”.

We apologize for this error.

